# *Drosophila* Morgana is an Hsp90-interacting protein with a direct role in microtubule polymerisation

**DOI:** 10.1242/jcs.236786

**Published:** 2020-01-23

**Authors:** Valeria Palumbo, Ammarah Tariq, Lori Borgal, Jeremy Metz, Mara Brancaccio, Maurizio Gatti, James G. Wakefield, Silvia Bonaccorsi

**Affiliations:** 1 Dipartimento di Biologia e Biotecnologie Sapienza, Università di Roma, 00185 Rome, Italy; 2 Biosciences/Living Systems Institute, College of Life and Environmental Sciences, University of Exeter, Exeter EX4 4QD, UK; 3 Dipartimento di Genetica, Biologia e Biochimica, Università di Torino, 10126 Torino, Italy; 4 Istituto di Biologia e Patologia Molecolari del CNR, 00185 Rome, Italy

**Keywords:** *Morgana*, Embryo mitosis, Spindle assembly, Hsp90, Microtubule binding, Microtubule polymerisation, *Drosophila*

## Abstract

Morgana (Mora, also known as CHORD in flies) and its mammalian homologue, called CHORDC1 or CHP1, is a highly conserved cysteine and histidine-rich domain (CHORD)-containing protein that has been proposed to function as an Hsp90 co-chaperone. Morgana deregulation promotes carcinogenesis in both mice and humans while, in *Drosophila*, loss of *mora* causes lethality and a complex mitotic phenotype that is rescued by a human *morgana* transgene. Here, we show that *Drosophila* Mora localises to mitotic spindles and co-purifies with the Hsp90–R2TP–TTT supercomplex and with additional well-known Hsp90 co-chaperones. Acute inhibition of Mora function in the early embryo results in a dramatic reduction in centrosomal microtubule stability, leading to small spindles nucleated from mitotic chromatin. Purified Mora binds to microtubules directly and promotes microtubule polymerisation *in vitro*, suggesting that Mora directly regulates spindle dynamics independently of its Hsp90 co-chaperone role.

## Abstract (precis)

**Summary:** Morgana, a *Drosophila* homologue of an oncogene, physically interacts with Hsp90 complexes and has an independent function in regulating microtubule polymerisation, both *in vitro* and *in vivo*.

## INTRODUCTION

Most eukaryotic proteomes include proteins containing tandemly arranged cysteine and histidine-rich domains (CHORDs) (Shirasu et al., 1999; Heise et al., 2007; Zhang et al., 2010). These domains are functionally associated with CHORD-containing proteins and Sgt1 (CS) domains, which can exist either within the same protein or in separate proteins. Two such mammalian proteins, Morgana (also known as CHORDC1 and CHP1) and Melusin (also known as ITGB1BP2), contain both CHORD and C-terminal CS domains (Brancaccio et al., 2003; Ferretti et al., 2010), interact with the cytosolic forms of the heat-shock protein Hsp90 (Hsp90α and Hsp90β, collectively referred to as Hsp90) and have been proposed to act as Hsp90 co-chaperones (Hahn, 2005; Wu et al., 2005; Sbroggiò et al., 2008; Ferretti et al., 2010; Gano and Simon, 2010; Michowski et al., 2010; Hong et al., 2013; Bohush et al., 2019). Hsp90 and its co-chaperones mediate protein conformation shifts during the cell cycle, ultimately controlling stability and degradation of more than 300 ‘client’ proteins (Sahasrabudhe et al., 2017; Schopf et al., 2017).

In mammalian cells, Morgana interacts with ROCK kinases, and Morgana deficiency increases ROCK2 activity (Ferretti et al., 2010; Fusella et al., 2014), leading to centrosome overduplication (Ma et al., 2006; Hanashiro et al., 2011; Ferretti et al., 2010). Morgana has been also implicated in cancer, and there is data to suggest that Morgana acts both as an onco-suppressor and a proto-oncogene (Ferretti et al., 2010; Brancaccio et al., 2015; Fusella et al., 2014, 2017).

We have previously shown that the *Drosophila morgana* homologue *mora* (also known as *CG6198* or *CHORD*) is essential and that animals homozygous for *mora*-null mutations die as third-instar larvae (Ferretti et al., 2010). Their proliferative brain cells exhibit defects in chromosome condensation, abnormal spindles, polyploidy and diploid cells with multiple centrosomes. These aberrant phenotypes are fully rescued by a human *morgana* transgene, demonstrating functional conservation between flies and humans, but their cause and the relationship, if any, to an Hsp90-related role for Mora, is unknown (Ferretti et al., 2010).

*Drosophila* embryos are an ideal system for both high-resolution microscopy (Hayward et al., 2014) and proteomics (Palumbo et al., 2015). Moreover, the possibility to perturb functions through mutation, *in vivo* RNAi or acutely, through interfering antibody injection, allows complex phenotypes to be dissected. We therefore turned to the *Drosophila* embryo to investigate the mitotic role of Mora and the functional relationship between Mora and Hsp90.

Here, we show that in embryos Mora interacts physically with Hsp90 and its associated co-chaperones, and that reduction of Mora function leads to defective chromosome condensation and spindle formation but not to centrosome amplification. We further show that Mora associates with the spindle *in vivo* and binds to MTs *in vitro*, stimulating MT polymerisation. Thus, although some of the phenotypic defects elicited by loss of Mora could result from disturbances in the Hsp90 machinery, our data suggest that Mora directly controls MT behaviour during spindle assembly.

## RESULTS AND DISCUSSION

### Mora associates with the embryonic mitotic spindle

**Fig. 1.**
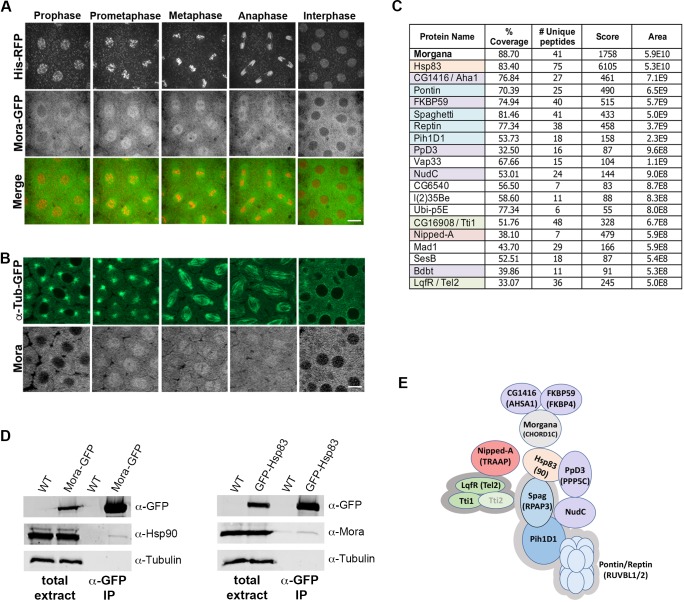
**Mora dynamically associates with embryonic spindles and co-purifies with Hsp90-related protein complexes.** (A,B) Stills from time-lapse videos of syncytial embryos expressing (A) Histone (His)–RFP (red) and Mora–GFP (green) or (B) α-Tubulin–GFP (green), injected with Alexa Fluor 633-conjugated anti-Mora antibody (greyscale) (see Movies 1 and 2; and Fig. 4 and Fig. S1). (C) Table of Mora-interacting proteins, isolated from early embryo extracts. Orange, Hsp family member; blue, R2TP complex member; green, TTT complex member; red, PIKK family member; purple, β-propeller-containing protein. (D) Western blots of anti-GFP IPs from control (WT) embryos, and embryos expressing either Mora–GFP or GFP–Hsp83. (E) Sketch of possible physical interactions between Hsp90, Mora and Mora interactors, based on the AP-MS and extant data from humans. The conserved human R2TP complex (RPAP3, Ruvbl1, Ruvbl2 and PIH1D1) (light grey outline) brings Hsp90 close to client proteins such as RNA polymerase II (Pol II) and phosphatidylinositol 3-kinase-related kinases (PIKKs). The PIKK enzymes – including TRAAP, the only PIKK devoid of protein kinase activity – interact with R2TP, and thereby Hsp90, through the TTT complex (Tel2–Tti1–Tti2) (dark grey outline). CHORDC1 also biochemically interacts with the Hsp90 co-interactors AHSA1, FKBP4 and PPP5C. PPP5C interacts with NudC, which itself associates with PIH1D1 (data from thebiogrid.org). Scale bars: 10 µm.

Following fertilisation, *Drosophila* embryos undergo 13 rapid, synchronous divisions within a syncytium, using proteins laid down by the mother (Foe and Alberts, 1983). To investigate the dynamic behaviour of Mora in living embryos, we generated a fly line carrying a UAS-*mora*-GFP construct. The encoded Mora–GFP protein is fully functional, and rescues the lethality of homozygous *mora* mutants (Ferretti et al., 2010). Time-lapse spinning-disc confocal microscopy demonstrated that Mora–GFP is primarily cytoplasmic during interphase, with an enrichment in the perinuclear area and a weak nuclear localisation (Fig. 1A; Fig. S1A, Movie 1). Upon nuclear envelope breakdown (NEB), Mora–GFP becomes enriched at mitotic spindles, remaining associated with spindle microtubules (MTs) throughout mitosis. This dynamic localisation is confirmed by the observation that an Alexa-Fluor-633-conjugated anti-Mora antibody localises similarly when injected into *Drosophila* embryos (Fig. 1B; Fig. S1B, Movie 2).

### Morgana interacts with the Hsp90–R2TP–TTT super-complex and other Hsp90 co-chaperones

To obtain insight into the cellular function of Mora, we sought to identify its interacting partners. Extracts from 0–3 h embryos expressing Mora–GFP were incubated with GFP–TRAP-A and subjected to affinity purification-mass spectrometry (AP-MS). Mora–GFP was efficiently purified (Fig. S1C) and, after applying stringent filtering against a database of non-specific interactors (Palumbo et al., 2015), was the most abundant hit found (Fig. 1C; Table S1). A second AP-MS experiment identified the same core interacting proteins with similar abundance, demonstrating the reproducibility of these interactions (Table S1).

Previous studies in mammalian cells have shown that Morgana and Hsp90 co-immunoprecipitate (Hahn, 2005; Wu et al., 2005; Ferretti et al., 2010; Gano and Simon, 2010; Michowski et al., 2010; Hong et al., 2013). This interaction is conserved, as the *Drosophila* homologue (Hsp83) was purified with similar abundance to Mora itself (Fig. 1C). To verify this interaction, we carried out a reciprocal co-immunoprecipitation (co-IP) using embryo extracts expressing either Mora–GFP or Hsp83–GFP (Fig. 1D).

Hsp90 is the hub of a chaperone machinery required for folding, stabilisation and activation of many proteins, which relies on evolutionarily conserved co-chaperones that act as adaptors, facilitating client recruitment (Biebl and Buchner, 2019). When we restricted our analysis of Mora-interacting proteins to those present at levels of ∼1:100 or greater than Mora itself, we found that 13 of the 18 interactors had conserved chaperone-related ontologies (Fig. 1C). These include seven of the eight members of the Hsp90–R2TP–TTT super-complex (Rivera-Calzada et al., 2017) and one of its target clients, Nipped-A (also known as TRRAP) (Fig. 1C,E). Five other interactors share homology with mammalian co-chaperones or with protein families that include co-chaperones; FKBP59 (also known as FKBP4), Bdbt (also known as TTC) and PPD3 (also known as PPP5C), which contain tetratricopeptide (TPR)-like domains, and NudC and CG16908 (also known as Aha1 and AHSA1), which are non-TPR co-chaperones that associate with β-propeller protein folds (Taipale et al., 2014; Biebl and Buchner, 2019) (Fig. 1C,E). Four of these have been demonstrated to physically interact with human Morgana (Fig. 1E; Taipale et al., 2014). Thus, *Drosophila* Mora is indeed likely to be an Hsp83 (Hsp90) co-chaperone.

### Morgana is required for early embryogenesis

**Fig. 2.**
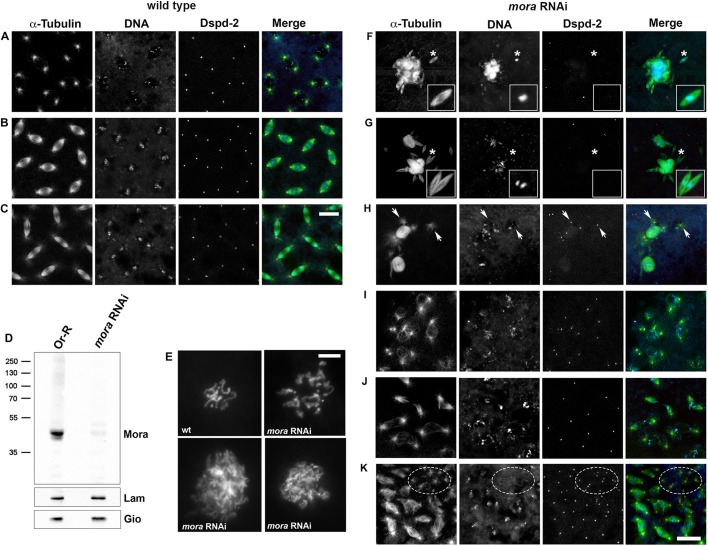
**Mora-depleted embryos exhibit defects in both chromatin condensation and spindle morphology.** (A–C) WT and (F–K) Mora-depleted 0–3 h embryos stained for Tubulin (green), the centrosomal marker DSpd-2 (red) and DNA (blue, DAPI). (A–C) Cells are in prophase (A), metaphase (B) and anaphase (C). (D) Western blotting of WT (Or-R) and *mora*^RNAi^ embryo extracts. The anti-Mora antibody recognises a band of the expected molecular mass, which is reduced in intensity by 88±4% in the *mora*^RNAi^ embryo compared to WT (mean±s.e.m. of five independent experiments). Giotto (Gio) and Lamin (Lam) are loading controls. (E) Metaphases in colchicine-treated WT (wt; diploid metaphase) and *mora*^RNAi^ embryos (RNAi; polyploid metaphases). (F–K) Mitotic defects in Mora-depleted embryos. (F,G) Chromatin aggregates associated with MTs and surrounded by small acentrosomal spindles (asterisks). Insets show enlargements of asterisk-marked regions. (H) Barrel-shaped spindles with detached centrosomes (arrows). (I) Aberrant mitotic figures with abnormally condensed chromosomes. (J) Anaphase-like figures with abnormally segregating chromosomes. (K) Blastoderm region with defective spindles and groups of free centrosomes (framed by a broken oval) generated by the sinking into the embryo interior of aberrant mitotic products. Scale bars: 10 µm (A–C, F–K); 5 µm (E).

To investigate the roles of Mora during embryo mitosis, we used RNAi to specifically reduce the Mora level in the female germline. In 0–3 h embryos from V32-Gal4 >UAS-*mora*-^RNAi^ mothers (*mora*^RNAi^ embryos; V32-Gal4 is specifically expressed in ovaries), Mora was strongly reduced (Fig. 2D). To exclude the possibility of off-target effects, we drove the same *mora* RNAi construct in neuronal cells using the elav-Gal4 driver. These RNAi animals died as third-instar larvae and their brains displayed a mitotic phenotype comparable to that observed in *mora* mutants (Ferretti et al., 2010) (Fig. S2).

*mora*^RNAi^ embryos failed to hatch (0/376; three independent experiments). To ascertain why, we stained 0–3 h fixed control and *mora*^RNAi^ embryos for DNA, Tubulin and the centrosomal marker Spd-2 (Giansanti et al., 2008). Wild-type embryos displayed nuclei that undergo mitosis synchronously, showing two Spd-2 signals at the spindle poles (Fig. 2A–C). In contrast, 33% (*n*=191) of *mora*^RNAi^ embryos did not show any sign of nuclear proliferation, while the remainder exhibited aberrant phenotypes, that fell into three broad categories. The first showed large chromatin aggregates not associated with centrosomes but surrounded by irregular masses of MTs (23%; *n*=191). These chromatin–MT clumps were located in the embryo interior and often accompanied by small but well-organised ‘satellite’ acentrosomal spindles (Fig. 2F,G). This observation indicates that *Drosophila* embryos can assemble well-focused spindles exploiting MTs nucleated near the kinetochores. To arrest the dividing nuclei in metaphase, we treated the embryos for 20 min with colchicine; in chromosome preparations from these embryos, we observed many polyploid figures, often showing abnormally condensed chromosomes (Fig. 2E). This suggests that the chromatin clumps in Mora-depleted embryos are generated by successive failures of chromosome segregation followed by re-entry into interphase.

The second category of aberrant phenotypes (19%; *n*=191) consisted of barrel-shaped spindles with an apparent metaphase configuration but showing two separated centrosomes at each pole, often detached from the spindles (Fig. 2H). These mitotic figures, located in the embryo interior, are the likely outcome of defects in mitotic spindle assembly, such as those caused by mutations in *dynein*, *asp*, *mars* (*HURP*) or *cfo* (Goshima et al., 2005; Morales-Mulia and Scholey, 2005; Zhang et al., 2009; Wakefield et al., 2000).

The third phenotypic category (25%; *n*=191) comprised embryos displaying patches of defective mitoses with irregularly condensed chromosomes and abnormally organised spindles (Fig. 2I) or severely aberrant anaphases (Fig. 2J). We also observed regions containing pairs of centrosomes still nucleating MTs but no longer associated with the chromatin (Fig. 2K); likely the consequence of ‘nuclear fall-out’, where abnormal nuclei from previous divisions are pulled into the embryo interior, leaving centrosomes at the embryonic cortex (Sullivan et al., 1990, 1993). Most embryos (93%) showed only one of the three broad phenotypic categories described above. A likely explanation for this observation is that these phenotypes depend on the developmental time at which the embryo was fixed.

Finally, in contrast to Mora-depleted brains, fixed *mora*^RNAi^ embryos did not exhibit centrosome amplification; there were no examples of multipolar spindles with more than two centrosomes, nor clusters of over-duplicated centrosomes at the embryo periphery.

### Acute inhibition of Mora in embryos perturbs spindle formation but has no effect on centrosome duplication

To understand the primary consequences on mitosis of Mora loss, we acutely perturbed Mora function through injecting anti-Mora antibodies directly into early embryos. We generated and affinity purified a rabbit antibody that reacts with a single protein of ∼40 kDa (the predicted Mora molecular mass) and showed a signal that was strongly reduced in *mora*^RNAi^ embryos (Fig. 2D) and colocalised with Mora–GFP (Fig. 1B; Fig. S1B, Movie 2).

**Fig. 3.**
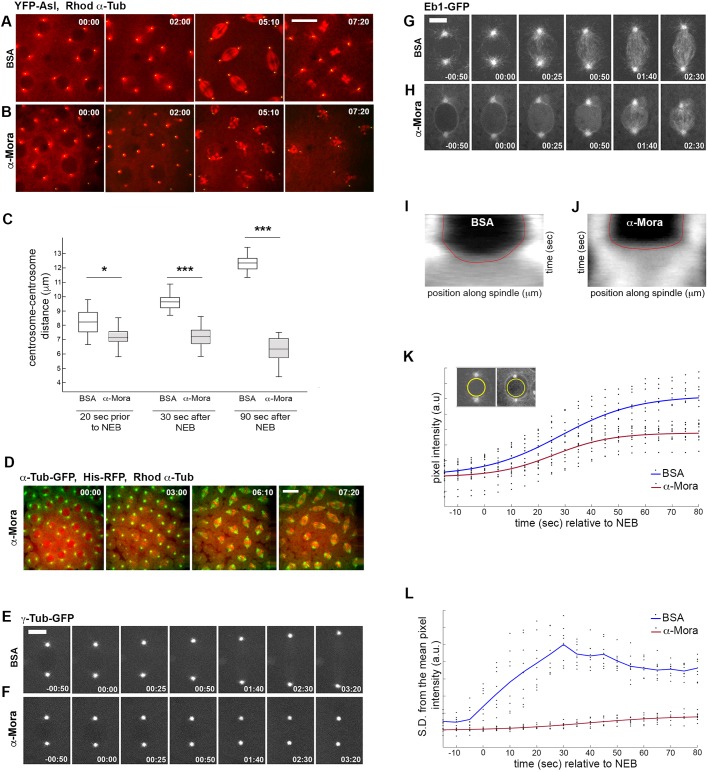
**Acute Mora inhibition causes a severe mitotic spindle phenotype.** (A,B,D–H) Stills from time-lapse videos of mitosis in embryos expressing spindle-associated proteins. The numbers indicate time (min:s) elapsed from the beginning of imaging. (A,B) YFP–Asl (green)-expressing embryos simultaneously injected with Rhodamine–Tubulin (red) and either BSA (control, A) or anti-Mora antibody (B) (Movies 3 and 4). (C) Quantification of centrosome–centrosome distance in BSA-injected or anti-Mora-injected embryos (*n*=25 spindles from 3 embryos). The box represents the 25–75th percentiles, and the median is indicated. The whiskers show the complete range. *P=0.005, ***P<0.00001 (one-tailed unpaired *t*-test). (D) Embryos expressing Histone (His)–RFP (red) and α-Tubulin–GFP (green) simultaneously injected with Rhodamine–tubulin (red) and anti-Mora antibodies (Movie 5). (E,F) Embryos expressing γ-Tubulin–GFP injected with BSA (E) or anti-Mora (F) exhibit comparable γ-Tubulin fluorescence at centrosomes. (G,H) Embryos expressing Eb1–GFP injected with BSA (G) or anti-Mora antibody (H). Anti-Mora injection strongly suppresses Eb1 comet dynamics (Movies 8 and 9). (I,J) Composite kymographs from the spindles in G and H, showing Otsu's threshold contours in red, differing between control- and anti-Mora-injected spindles. Total distance (*x* axis), 14 μm; total time (*y* axis), 200 s. (K) Eb1–GFP fluorescence intensity over time within the nuclear region dived by the spindle region in control- (blue) and anti-Mora-injected (red) embryos. *n*=9 for each condition. (L) The standard deviation of the Eb1–GFP fluorescence intensity as shown in K. Time 00:00 indicates NEB. Scale bars: 10 µm (A,B,D); 5 µm (E–H). a.u., arbitrary units.

We first analysed the effects of acute Mora inhibition by simultaneously injecting anti-Mora antibodies and Rhodamine-labelled Tubulin (Rhod–Tubulin) into embryos expressing YFP–Asterless (YFP–Asl) (Rebollo et al., 2007), to follow both MTs and centrosomes. Consistent with previous work (for example, Hayward et al., 2014), bovine serum albumin (BSA)-injected control embryos formed regular mitotic spindles (Fig. 3A; Movie 3). In contrast, anti-Mora-injected embryos displayed normal centrosomes but, upon nuclear envelope breakdown (NEB), formed disorganised spindles (Fig. 3B; Movie 4). These spindles were shorter than in BSA-injected embryos, as shown by the significantly reduced centrosome-to-centrosome distance during spindle formation (Fig. 3C). To confirm these results, we injected embryos co-expressing α-Tubulin–GFP and Histone 2AV–RFP with both anti-Mora antibodies and Rhod–Tubulin (Fig. 3D), imaging at lower magnification to follow mitosis both in the region near to the site of injection (marked by high Rhodamine fluorescence) and further away (serving as a control). Within these embryos, prophase asters and gross centrosome morphology appeared normal. However, upon NEB, in regions of intense Rhod–Tubulin and anti-Mora fluorescence, the spindles showed delayed assembly and abnormal morphologies, and were substantially shorter than those outside the area of Rhod–Tubulin fluorescence (Fig. 3D; Movie 5). These malformed spindles were insufficient for effective chromosome segregation, resulting in abortive anaphase figures with lagging chromatin masses (Fig. 3D; Movie 5). As expected, the products of these highly irregular divisions sank into the embryo interior (Sullivan et al., 1990, 1993) (Fig. 3D; Movie 5).

Consistent with the analysis of fixed *mora*^RNAi^ embryos, but in contrast to *mora* mutant or *mora*^RNAi^ brain cells (Fig. S2), injection of anti-Mora antibodies into embryos did not result in centriole overduplication (Fig. S3, Movies 6 and 7). This probably reflects tissue-dependent roles of Mora in centriole duplication, as observed for other proteins involved in this process; for example, DSas-6 overexpression drives centriole amplification in embryos, but has no effect on spermatocyte centrioles (Peel et al., 2007).

### Acute inhibition of Mora dramatically reduces centrosomal MT stability

To obtain further insight into the role of Mora in spindle assembly, we assessed the dynamic localisation of both γ-Tubulin and the MT plus-end protein Eb1 (Piehl et al., 2004). In both control- and anti-Mora-injected embryos, we saw no qualitative difference in the recruitment of γ-Tubulin to centrosomes (Fig. 3E,F), suggesting that Mora inhibition does not affect the ability of the centrosomes to nucleate MTs. However, a consistent and dramatic difference in Eb1–GFP localisation was observed. We have previously demonstrated that, in *Drosophila* embryo mitoses, Eb1–GFP is present as fluorescent ‘comets’ at the growing plus-ends of MTs (Hayward et al., 2014). In control-injected embryos, Eb1–GFP comets emanated radially from the centrosomes and, at NEB, moved towards the spindle equator (Fig. 3G; Movie 8). In anti-Mora-injected embryos, although Eb1–GFP accumulated at centrosomes in prophase, upon NEB there was no Eb1 radial expansion from centrosomes and comets were absent in the nuclear space; instead, fluorescence in the nucleus appeared to increase uniformly, suggestive of a transient increase in nucleoplasmic, rather than MT-associated, Eb1 (Fig. 3H; Movie 9). Over time, MT-associated Eb1–GFP accumulated in the peri-chromosomal region. However, Eb1–GFP fluorescence was much weaker than in controls and only small, ill-defined Eb1–GFP comets appeared to form.

To quantitatively assess Eb1–GFP behaviour in control- and anti-Mora-injected embryos, we measured three parameters. First, we used our previously described image analysis software (Hayward et al., 2014) to generate composite kymographs of Eb1–GFP fluorescence across spindle length over time, calculating the fluorescence intensity contour, based on Otsu's threshold algorithm (see Materials and Methods). In control embryo kymographs, the gradient of the threshold demonstrated the Eb1–GFP comet movement over time from centrosomes to the spindle equator (Fig. 3I). In contrast, in anti-Mora-injected embryos the threshold gradient was essentially flat (Fig. 3J). Second, we measured the mean Eb1–GFP fluorescence from 15 s before to 80 s after NEB, in a circle just smaller than the nucleus (Fig. 3K). The mean fluorescence in both control- and anti-Mora-injected nuclei increased following NEB, as the mature spindle formed, though it was substantially greater in control nuclei than in Mora-inhibited nuclei (Fig. 3K). Finally, we calculated the standard deviation from the mean fluorescence in the nucleus divided by that in spindle region over time. In control nuclei, this value dramatically increased following NEB, as Eb1–GFP comets entered the nuclear space, and decreased as the mature spindle formed and Eb1–GFP fluorescence filled the circle (Fig. 3L). In contrast, in anti-Mora-injected embryos, the standard deviation remained low after NEB. Together, these measurements confirm the different dynamics of Eb1–GFP behaviour, indicating that following Mora inactivation, centrosomally derived MT plus-end dynamics are substantially inhibited.

### Morgana directly binds MTs and stimulates MT polymerisation *in vitro*

**Fig. 4.**
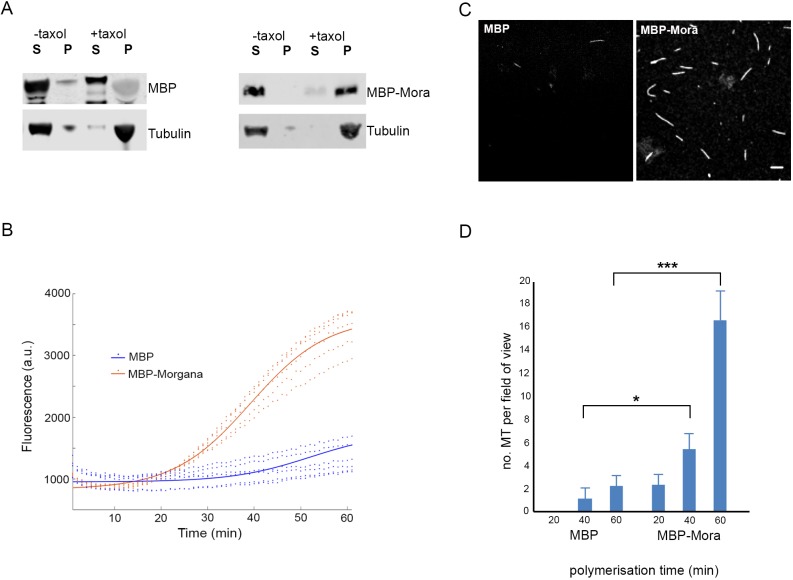
**Mora binds MTs and increases MT polymerisation *in vitro*.** (A) Western blots of *in vitro* MT co-sedimentation assays. Without taxol (−), Tubulin, MBP and MBP–Mora are in the supernatant (S); with taxol (+), polymerised Tubulin sediments in the pellet (P) with MBP–Mora, but not with MBP. (B) Graph of Tubulin polymerisation assays, where fluorescence is directly related to the amount of Tubulin polymer present. The curves are a sigmoidal fit to six data points (dots) for three independent purification experiments, each undertaken in duplicate. (C) Confocal images of fixed, fluorescent polymerisation assays at *t*=60 min for each condition. (D) Quantification and statistical significance of the number of MTs per field of view under the polymerisation conditions described in B (*n*=10 fields of view per condition). *P<0.05, ***P<0.00001 (one-tailed unpaired *t*-test).

Our analyses suggest a role for Mora in regulating MT dynamics *in vivo*. To substantiate this, we asked whether pure Mora can regulate MT growth *in vitro*. Bacterially expressed and purified full-length Mora, fused to maltose-binding protein (MBP), ran as two closely associated bands on SDS-PAGE, each of which was identified by western blotting with anti-Mora and anti-MBP antibodies (Fig. S4). MT co-sedimentation assays demonstrated that MBP–Mora, but not MBP alone, specifically bound to MTs *in vitro* (Fig. 4A).

Next, we asked whether MBP–Mora can stimulate MT polymerisation. Pure MBP or MBP–Mora were incubated with Tubulin and GTP at 37°C, and polymerisation was assessed via a fluorescence assay (see Materials and Methods). While MBP alone was not capable of stimulating MT growth under these conditions, incubation with MBP–Mora resulted in reproducible and robust MT polymerisation (Fig. 4B). In addition, we fixed and stained the MTs generated by the polymerisation assays, and quantified the number of MTs under a fluorescence microscope (Fig. 4C). Incubation with MBP–Mora generated many more MTs than incubation with MBP alone (Fig. 4D). Collectively, these results strongly suggest that Mora is capable of stimulating MT polymerisation *in vitro*.

### The mitotic roles of Mora

*Drosophila* Mora, like its human counterpart, is an Hsp90-interacting protein that co-precipitates with many other co-chaperones. We found that Mora deficiency leads to defects in chromosome condensation and spindle formation in both embryos and larval brains. These defects could result from disturbances in the Hsp90 machinery, as Hsp90 and several of its co-chaperones have been implicated in aspects of mitosis (Lange et al., 2000; Gartner et al., 2003; Li et al., 2004; Ducat et al., 2008; Fielding et al., 2008; Somma et al., 2008; Gentili et al., 2015). However, the results described here are consistent with an Hsp90-independent role of Mora in regulating mitotic MT dynamics. We do not yet have a molecular understanding of the relationship between Mora and MTs *in vivo*. It is possible that Morgana functions in spindle assembly through interacting MAPs. Gene Ontology (GO) enrichment analysis for the Mora-interacting proteins identified via MS did highlight the term ‘microtubule cytoskeleton organisation’ as slightly enriched (2×10^−3^), identifying Mora itself and seven interactors; Cdk2, Mad2, Hsp83, ProsBeta5, Mora, Vap33 and Pontin (Table S1). Of these, Pontin has previously been described in a *Drosophila* RNAi screen for mitotic spindle regulators, where it was shown to interact functionally with the γ-Tubulin ring complex (Ducat et al., 2008). However, reduction of Pontin resulted in reduced centrosomal γ-Tubulin localisation (Ducat et al., 2008), whereas inhibition of Morgana in embryos results in astral MT instability without affecting centrosomal γ-Tubulin levels. Moreover, in support of a direct relationship between MTs and Mora, we have shown that purified MBP–Mora is able to bind to MTs and stimulate MT nucleation and/or polymerisation *in vitro*. Thus, we favour the hypothesis that the abnormal spindle phenotypes seen in embryos and brains primarily result from a direct effect of Mora deficiency on MT behaviour.

## MATERIALS AND METHODS

### *Drosophila* strains and husbandry

The fly stocks bearing a PBac{PB}CHORDc02881 insertion (*mora^1^*) (Bloomington stock no. 11130; Ferretti et al., 2010) and expressing dsRNA for mora/*CHORD* RNAi under UAS control from the TRIP stock collection (stock no. 66324; Perkins et al., 2015) were both provided by the Bloomington Stock Center (Indiana University, Bloomington, IN). The line expressing an Hsp83–GFP transgene under the control of its endogenous promoter was a gift from Renato Paro (Department of Biosystems Science and Engineering, ETH Zürich, Switzerland) (Tariq et al., 2009). To obtain the inducible Mora–GFP fusion, the *CG6198* gene was amplified from an embryonic cDNA library using primers 5′-CACCATGGAACAATGCTATAAC-3′ and 5′-ATCTAAGTTGTTTGGGCT-3′ that span the entire open reading frame, and was cloned into pPWG vector via pENTR/D-TOPO (Invitrogen). The plasmid was injected into *w1118* embryos by Bestgene Inc (Chino Hills, CA), using standard procedures. Both the silencing and the expression in the female germline was achieved by combining flies carrying a single copy of a specific transgene with a copy of a *matα-Tubulin V32-GAL4* (a gift from Monica Bettencourt-Dias, Instituto Gulbenkian de Ciência, Portugal). Mutant phenotypes in brains (Ferretti et al., 2010) were obtained inducing RNAi with an elav-GAL4 driver (Bloomington stock no. 25750). For live embryo analysis, we used fly stocks expressing the following fluorescent proteins: α-Tubulin–GFP and His2Av–mRFP (Hayward et al., 2014); YFP-Asl [gift from Cayetano Gonzalez, Institute for Research in Biomedicine (IRB Barcelona), Spain; Rebollo et al., 2007]; γ-Tubulin-GFP and Eb1-GFP (gifts from Sharyn Endow, Department of Cell Biology, Duke University Medical Center, USA; Hallen et al., 2008; Liang et al., 2009). Oregon R (Or-R) was used as control strain. All flies were reared according to standard procedures and maintained at 25°C. The genetic markers and special chromosomes are described in detail in FlyBase (http://www.flybase.org). To determine the embryonic lethality phase, 4-day-old females of the suitable genotype were crossed to Or-R homozygous males. Eggs were collected from fertilised females using apple-grape juice agar plates. The eggs were monitored for several days and any first instar larvae were counted and transferred to fresh culture for further development, as previously described (Tritto et al., 2015).

### Expression and purification of MBP–Mora

The full coding sequence of Mora was PCR amplified, sequenced and subsequently EcoRI/BamHI cloned into pMAL-c2x vector (New England Biolabs). The construct, or pMal alone, was transformed into BL21 (DE3) cells. Individual colonies were grown at 37°C until they reached an optical density at 600 nm (OD_600_) of 0.4–0.6, before addition of IPTG to a final concentration of 0.1 mM and further growth for 4 h. Cells were harvested by centrifugation (6000 ***g*** for 10 min), resuspended in 20 mM Tris-HCl (pH 8.0) with 100 mM NaCl with cOmplete EDTA-free Protease Inhibitor Cocktail (Roche) and disrupted by sonication. The resulting extract was clarified by centrifugation at 24,000 ***g*** for 30 min at 4°C to pellet cell debris and loaded onto an MBP Trap 1 ml affinity column (GE Healthcare) equilibrated with buffer A (20 mM Tris-HCl, 200 mM NaCl and 1 mM EDTA pH 7.4) using an ÄKTApure system. The fusion protein, or MBP alone, was eluted with buffer A plus 10 mM maltose. Fractions of 1.5 ml were collected, analysed by SDS-PAGE using a 10% polyacrylamide gel, and those containing MBP or MBP–Mora were pooled and applied onto a Hi-load Superdex *200 prep* grade column (Pharmacia Biotech) equilibrated in 20 mM HEPES, 500 mM NaCl. Fractions containing MBP or MBP–Mora were pooled, concentrated if necessary, and stored with 20% (v/v) glycerol at −20°C.

### Antibody generation

The polyclonal antibodies against Mora and the MBP were produced using MBP and MBP-Mora as immunogens. MBP–Mora and MBP were produced in *E. coli* using the pMAL Protein Fusion and Purification System (New England BioLabs) and affinity purified on amylose resin columns. Rabbits were immunised by repeated intramuscular injections of the purified fusion proteins (500 μg) suspended in Complete Freund Adjuvant. The specificity of the antiserum against Mora was tested by western blotting on protein extracts from wild-type and *mora* mutant flies. The specificity of the antiserum against MBP was tested by western blotting on protein extracts from *E. coli* expressing or not expressing MBP. The anti-Mora antibodies were affinity-purified from rabbit serum using a Sepharose column coupled to glutathione S-transferase (GST)–Mora produced in *E.coli* using the GST Gene Fusion System (GE Healthcare Life Sciences) and eluted with glycine-HCl (pH 3). The anti-MBP antibodies were purified using the same protocol on a Sepharose column coupled to MBP.

### Affinity purification and analysis of MS data

For GFP–TRAP-A isolation, ∼0.4 g of 0–3-h-old embryos, laid by females expressing full-length Mora–GFP under the control of the maternal driver *V32-GAL4* were homogenised in 1.5 ml of C buffer (50 mM HEPES pH 7.4, 50 mM KCl, 1 mM MgCl_2_, 1 mM EGTA, 0.1% IGEPAL CA-630) with protease inhibitors (Roche). Extract was clarified through centrifugation at 10,000 ***g*** for 10 min, 100,000 ***g*** for 30 min, and 100,000 ***g*** for a further 10 min. Clarified extract was incubated with 15 μl equilibrated GFP–TRAP-A beads (Chromotek) for 2 h at 4°C. The beads were then washed four times with ice-cold C buffer, stored at −20°C and processed for mass spectrometry as described in Palumbo et al. (2015). MS results were filtered by removing protein IDs with (i) <3 unique peptide hits, (ii) <20% peptide:protein coverage and (iii) overall MS scores of <50. These were run through our false-positive database, accumulated from eight independent control GFP–TRAP-A experiments (Palumbo et al., 2015). Any protein ID that was either not identified in negative control list or was identified in negative controls with MS Sscores of at least 1.5-fold less than in Mora–GFP was kept, while all other protein IDs were discarded.

### Cytology

For immunofluorescence experiments, 0–3-h-old embryos were collected at 25°C on agar plates and dechorionated in 50% bleach. After removal of the vitelline membrane in a mixture of methanol and heptane (1:1), embryos were fixed for 30 min in 3.7% formaldehyde in PBS under gentle agitation at room temperature and blocked for 1 h in 0.3% Triton X-100 PBS with 3% BSA before staining. For double immunostaining, whole-mount embryos were incubated overnight at 4°C with the following primary antibodies: rabbit anti-DSpd-2 (1:3500; Giansanti et al., 2008), monoclonal anti-α-Tubulin (1:1000; cat. no T6199, Sigma-Aldrich), which were detected by 1 h incubation at room temperature with Cy3-conjugated anti-rabbit IgG (1:300; Invitrogen) and fluorescein isothiocyanate (FITC)-conjugated anti-mouse IgG+IgM (1:20; Jackson Laboratories). Embryos were stained with TOTO-3 DNA dye (1:1000; Life Technologies) for 10 min at room temperature and then mounted in Vectashield medium H-1000 (Vector Laboratories). Confocal analysis was performed with a laser-scanning inverted microscope Zeiss LSM 780 (Zeiss, Oberckochen, Germany) equipped with a 63×/1.4 NA Oil Plan-Apochromat objective. Image acquisition and processing were achieved using the Zeiss Efficient Navigation (ZEN) software. The images shown are the maximum-intensity projections of optical sections acquired at 0.5 μm steps. Mitotic spreads of embryonic nuclei were performed according to Gao et al. (2009). Larval brains were fixed and stained according to Mengoli et al. (2014). Immunostained brains were mounted in Vectashield medium H-1200 (Vector Laboratories) containing the DNA dye DAPI, and examined with a Zeiss Axioplan fluorescence microscope equipped with a CCD camera (Photometrics CoolSnap HQ).

### *In vivo Drosophila* imaging, microinjection and image analysis

For *in vivo* time-lapse imaging, 1–2-h-old embryos expressing Mora–GFP and HisH2Av–mRFP were manually dechorionated and aligned in heptane glue on 22×50 mm coverslips, covered with a 1:1 mixture of Halocarbon oil 700 and Halocarbon oil 27 (Sigma), and imaged for a time series. For immunodepletion experiments, affinity purified anti-Mora antibodies were exchanged into injection buffer (100 mM HEPES, pH7.4 and 50 mM KCl), concentrated to 2-5 mg/ml, centrifuged at 13,500 ***g*** for 20 min and injected into cycle 10–11 embryos (previously desiccated for 6 min) expressing suitable fluorescently tagged proteins. For co-imaging of microtubules, immunodepleted embryos were co-injected with X-Rhodamine-labelled Tubulin (Cytoskeleton Inc.) at 5 mg/ml in injection buffer. As a control, embryos were injected with BSA (Sigma) dissolved into injection buffer at 5 mg/ml. To assess the dynamic localisations of Mora, affinity-purified antibodies at 2–5 mg/ml were first labelled with the Alexa Fluor™ 633 Protein Labeling Kit (Thermo Fisher Scientific), following the manufacturer's instructions. Injections were performed using an Eppendorf Inject Man NI two and Femtotips II needles (Eppendorf). Embryos were injected at the midpoint of their ventral side. At least five independent injections were performed for each experiment shown. Imaging was performed using a Visitron Systems Olympus IX81 microscope equipped with a CSO-X1 spinning disk using a UPlanS APO 1.3 NA (Olympus) 60× objective. Five 1-μm slice stacks were acquired with a 400 ms exposure per slice, at a constant room temperature of 22°C. Image processing and analysis was performed using ImageJ to produce accumulated projections. All videos are projections of 5 confocal sections 1.0 μm apart, recorded every 5 s; Movies 1 and 2 are presented at 10 frames per second, and Movies 3–9 to 7 at frames per second. Automated spindle tracking and kymograph generation was performed using custom image processing and object tracking algorithms, as detailed in Hayward et al. (2014). Composite kymographs shown are from single embryos, generated from between six and ten spindles. To aid in the analysis of the information contained in the kymograph data, we performed Otsu's threshold algorithm on a lightly Gaussian filtered (sigma=3.0) version of the kymographs. The resultant threshold value was used to plot the contour evaluated at this level through the filtered data, which provides a spatio-temporal growth profile of the MTs. To calculate the mean intensity fluorescence and standard deviation from the mean intensity of fluorescence in BSA- or anti-Mora-injected embryos expressing Eb1–GFP, a circular region corresponding to the interior of the nucleus was manually drawn in ImageJ and fluorescence intensity measured over 20 time points, each of 5 s, from just prior to NEB onwards. Ten nuclei and spindles were tracked for each condition.

Image processing and analysis was performed with FIJI software. Fluorescence loss caused by bleaching was corrected using the Bleach Corrector macro (developed by Kota Miura, EMBL Heidelberg, Germany). Time-lapse movies were generated of maximum intensity projections of time frames with levels adjusted to reduce background fluorescence. Measurement of cell cycle timings was undertaken manually. NEB to initial chromosome alignment was defined as the time taken from the first frame showing fluorescent protein influx into the nucleus to the frame where the condensed chromosomes reached a local maximum X:Y ratio. Spindle length (centrosome-to-centrosome) comparisons were undertaken by manually measuring a line from the centre of each pair of centrosomes in 25 spindles from BSA-injected and anti-Mora-injected embryos, expressing YFP–Asl (Fig. 3C). Values were plotted in Excel (Microsoft), box-and-whisker plots generated on-line in ALCULA and statistical significance (unpaired *t*-test) of different conditions on centrosome-centrosome length was calculated. A similar analysis, with similar conclusions, was undertaken for 25 spindles from BSA-injected and anti-Mora-injected embryos expressing GFP–γ-Tubulin (data not shown). To assess the fluorescence intensity and the standard deviation from the mean fluorescence intensity, a circle of circumference just smaller than the nucleus was drawn using the circle tool in FIJI and measurements were taken from nine BSA-injected and nine anti-Mora-injected spindles, for each time point. The mean intensity and mean of the standard deviation from the mean intensity were plotted using MATLAB.

### *In vitro* microtubule co-sedimentation assay

MBP or MBP-Mora were spun at 100,000 ***g*** for 15 min in an Optima MAX ultracentrifuge (Beckman) to remove any insoluble material. Then, 10 µl of the sample was incubated for 15 min at 37°C in general tubulin buffer (GTB; Cytoskeleton Inc.) with 2.25 mg/ml 99% pure bovine tubulin (Cytoskeleton Inc.) and 1 mM GTP. The sample was incubated for a further 10 min at 37°C in the presence of 100 µM taxol (+). A negative control (−) was run in parallel, with an incubation temperature of 4°C and taxol replaced with GTB. The samples were layered onto a cushion of GTB with 40% glycerol and centrifuged at 100,000 ***g*** for 45 min at 4°C. The pellet and supernatant fractions were collected individually, and proteins present in each fraction were determined by western blot analysis. The supernatant was collected and an equal volume of buffer used to resuspend the pelleted microtubules and interacting protein. Equivalent volumes of supernatant and pellet were run side-by-side using SDS-PAGE, and analysed by western blotting using anti-MBP (NEB) and anti-α-Tubulin antibody (DM1A; Sigma-Aldrich).

### MT polymerisation assays

MT polymerisation assays were performed using a fluorescence-based Tubulin polymerisation kit (Cytoskeleton Inc, Denver CO, cat. no. BK011P) following the manufacturer's instructions. Briefly, 5 μl of control buffer [BRB80 (80 mM K-PIPES pH 6.8, 1 mM EGTA and 1 mM MgCl_2_)] or protein sample was added into each well of the assay plate at the indicated concentrations after pre-warming the plate at 37°C for 1 min. Then, Tubulin solution (50 μl) was dispensed rapidly into each well. The polymerisation dynamics of Tubulin were monitored for 60 min at 37°C by measuring the change in fluorescence every 1 min using a TECAN infinite 200pro fluorimeter. The excitation was 350 nm, and the emission was 440 nm. Data presented in Fig. 4 are the summation of three independent polymerisation assays, each undertaken in duplicate wells (six data points). Polymerisation data sets were fitted using a sigmoidal function in MATLAB. This characterised each data set in terms of the maximum and minimum fluorescence, the *x* value (minimum) at 50% distance between maximum and minimum *y* fluorescence value and the slope. The code and data can be found at https://github.com/elifesciences-publications/microtubules. To visualise the polymerised MTs, Tubulin -polymerisation assays were performed as described above, but with the addition of Rhodamine–tubulin (Cytoskeleton, Inc., Denver, CO) at a 1:10 ratio with unlabelled porcine tubulin (final tubulin concentration, 2 mg/ml). At *t*=20, 40 or 60 min, 1 µl of polymerisation sample was added to 200 µl glutaraldehyde (0.5% final concentration in GTB plus 1 mM GTP) and incubated at room temperature for 15 min. Then, 1 µl of these fixed MTs were spotted onto glass coverslips and imaged. Statistical significance (unpaired *t*-test) of the presence of MBP–Mora, in comparison to MBP alone, on the number of MTs polymerised was calculated.

### Western blotting

For immunoblotting, embryos were homogenised in C buffer [50 mM HEPES pH 7.4, 50 mM KCl, 1 mM MgCl_2_, 1 mM EGTA, 0.1% IGEPAL CA-630, protease inhibitors (Roche)]. Extracts, clarified through centrifugation at 10,000 ***g*** for 10 min, and 100,000 ***g*** for 30 min, were run on standard SDS-PAGE gels, blotted and incubated with the following primary antibodies: rabbit polyclonal anti-Mora (1:10,000); mouse anti-GFP (1:10,000; Roche); mouse anti-Lamin Dm0 (1:5000; DSHB, ADL101 clone); rabbit anti-Giotto (1:4000; Vernì et al., 2004). For detection, the following HRP-conjugated secondary antibodies were used: anti-mouse IgG (Sigma) and anti-rabbit-IgG (GE Healthcare), diluted 1:5000–1:10,000. Samples were visualised by using enzyme-linked chemiluminescence and X-ray film, or imaged using an ECL detection kit (GE Healthcare). Band intensities were quantified by densitometric analysis with Image Lab software (Bio-Rad).

## Supplementary Material

Supplementary information
