# Supplementary material for: Drosophila Morgana is an Hsp90-interacting protein with a direct role in microtubule polymerisation
Source: J Cell Sci. 2020 Jan 23;133(2):jcs236786. doi: 10.1242/jcs.236786 (PMC6983718; doi:10.1242/jcs.236786)
Supplement: Supplementary information [file joces-133-236786-s1.pdf]

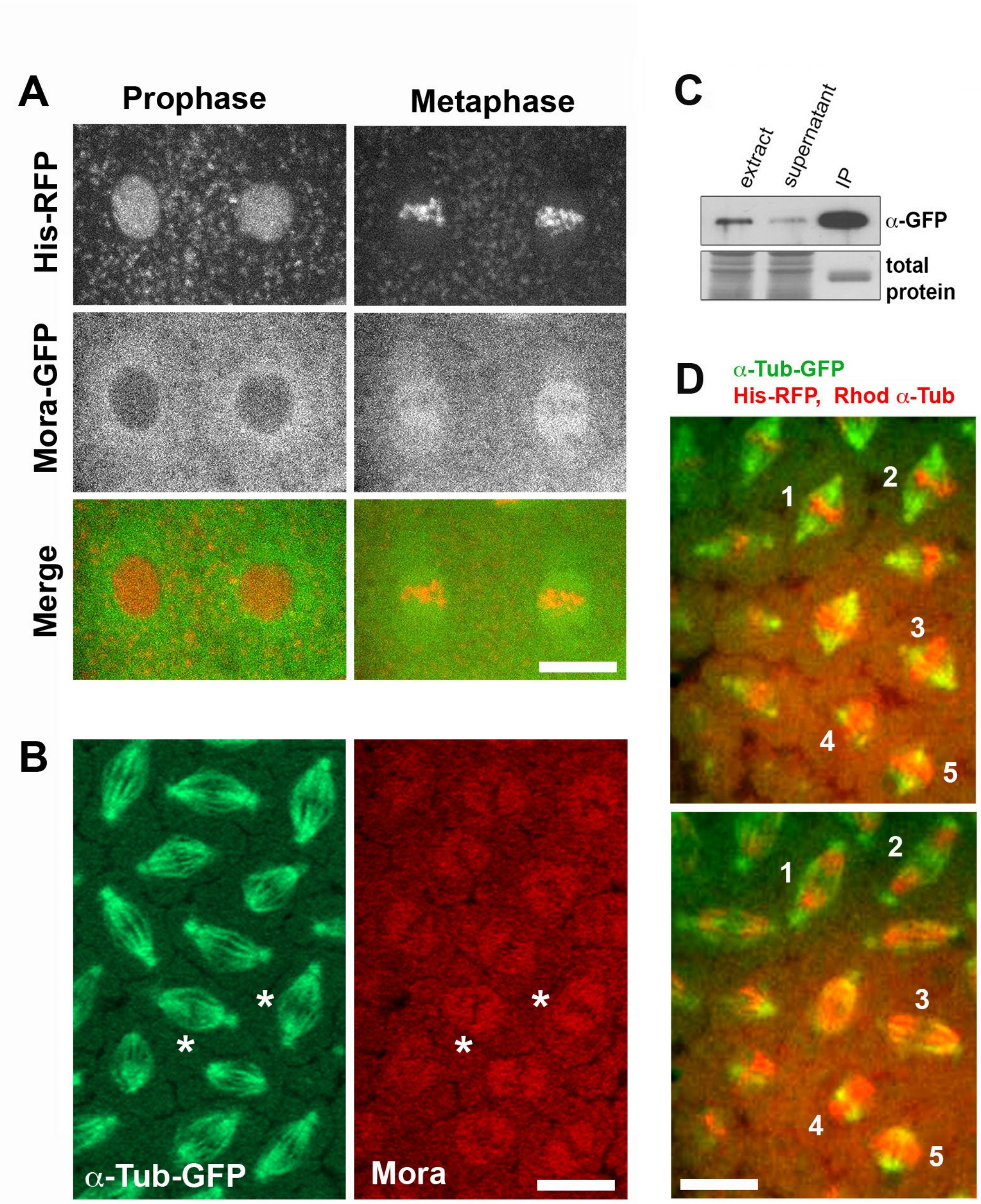

Figure S1. Mora localizes to mitotic spindles and anti-Mora antibodies disrupt spindle morphology. (A) Enlarged stills (from Figure 1A) of time-lapse videos of syncytial embryos expressing Histone-RFP (red) and Mora-GFP (green). Note the enrichment of Mora in the perinuclear areas of interphase nuclei and in metaphase spindles. (B) Enlarged stills (from Figure 1B) of time-lapse videos of embryos expressing Tubulin-GFP (green) injected with an Alexa 633-conjugated anti-Mora (red). Note the antibody binding to prometaphase/metaphase spindles. The asterisks mark two metaphase spindles. (C) Western blot of the Mora-GFP IP, used for AP-MS. Total protein is shown from a Coomassie-stained gel. (D) Enlarged stills (from Figure 3C) of time-lapse videos of embryos expressing both Histone-RFP (red) and α-tubulin-GFP (green) simultaneously injected with rhodamine-tubulin (used as a marker for antibody diffusion; red) and anti-Mora. Note the defects in spindle formation in the region marked by rhodamine-tubulin (spindles 3,4 and 5) while spindles outside of this region (spindles 1 and 2) appear normal. Scale bar, 10 µm. Related to Figures 1 and 3.

Journal of Cell Science • Supplementary information

Supplementary Figure S2

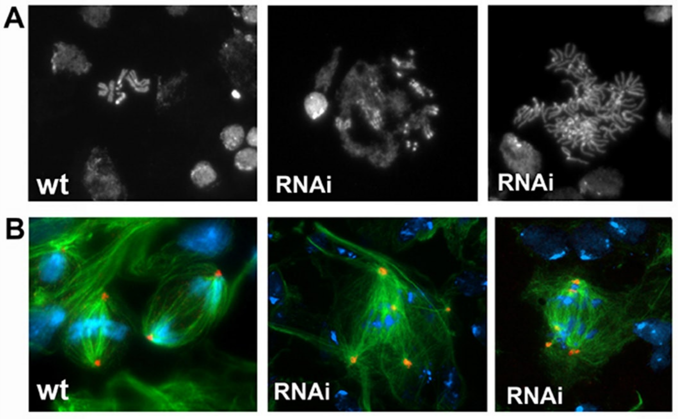

Figure S2. RNAi-induced Mora depletion causes severe mitotic defects in larval brains (A) Fixed larval brains from wild type controls (wt) and animals bearing a UAS-*mora*-RNAi construct in combination with the elav-Gal4 driver (RNAi). (i) DAPI stained brains. (ii) Brains stained for tubulin (green), the centrosomal marker DSpd-2 (red), and DNA (DAPI, blue). Note that Mora-depleted brains exhibit severe defects in chromosome condensation and polyploid cells (i), as well as disorganized spindles and dividing diploid cells with multiple centrosomes (ii). Scale bar, 5 μm. Related to Figure 2.

Journal of Cell Science • Supplementary information

Figure S3

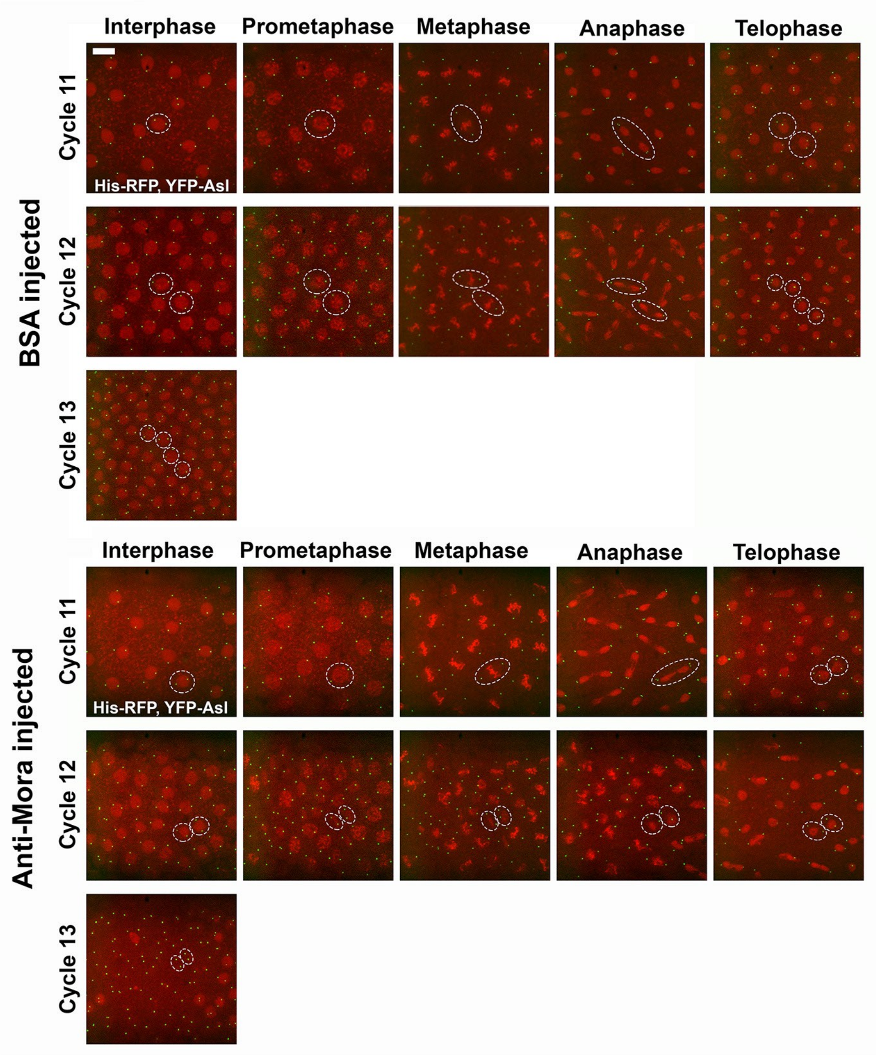

Figure S3. Acute Mora inhibition in embryos does not affect centrosome duplication

Stills from time-lapse videos of mitosis in GFP-Asl (green) and Histone-RFP (red) expressing embryos injected with either BSA or an anti-Mora antibody. Embryos were filmed from the 11th to the 13th cycle to assess the effect of acute Mora inhibition on centrosome duplication (Videos 6 and 7). Dashed circles identify a specific nucleus and its descendant nuclei during two division cycles. In the anti-Mora injected embryo each of the two nuclei resulting from the initial mitotic division possesses a couple of centrosomes. These nuclei fail to undergo the subsequent mitosis and at the beginning of the 13th cycle sink into the embryo interior. Note that Mora-inhibited embryos do not exhibit centrosome overduplication. Scale bar, 10 μm. Related to Figures 3 and 4.

Supplementary Figure S4

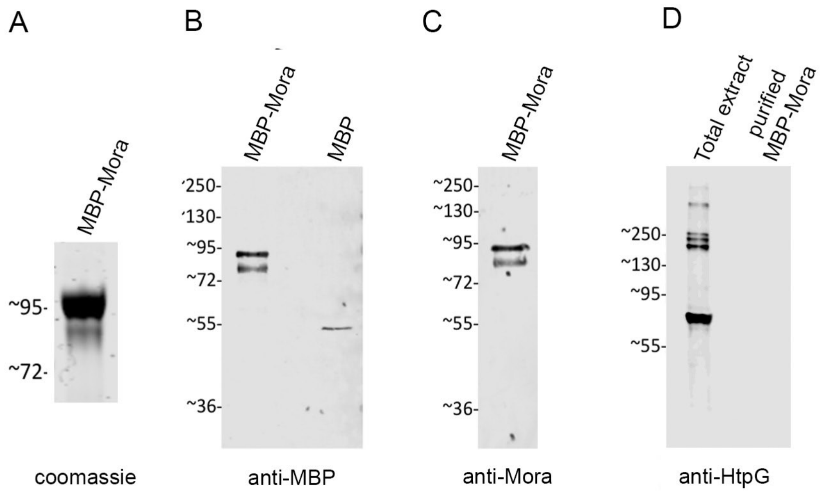

Figure S4. Purification of MBP and MBP-Mora

(A) Coomassie Brilliant Blue stained SDS-PA gel with purified MBP-Mora. (B) Western blot of purified MBP and MBP-Mora probed with anti-MBP antibodies. (C) Western blot of purified MBP-Mora probed with anti-Mora antibodies. (D) Western blot of total bacterial extracts and purified MBP-Mora, western blotted for the bacterial Hsp90 homologue, HtpG. HtpG is not co-purified. Related to Figure 4.

Table S1. Full MS datasets from two independent Morgana-GFP AP-MS experiments. Batches of ~0.4g of 0-3 hr embryos expressing Morgana-GFP were subjected to affinity purification using GFP-TRAP-A beads, followed by Mass Spectrometry. MS results were filtered by removing protein IDs with (i) <3 unique peptide hits, (ii) <20% peptide:protein coverage and (iii) overall MS Scores of <50. These were run through our false-positive database, accumulated from eight independent control GFP-TRAP-A experiments (Palumbo et al., 2015). Any protein ID that was either not identified in negative control list or was identified in negative controls with MS Scores of at least 1.5-fold less than in Morgana-GFP was kept, while all other protein IDs were discarded. The colour scheme refers to Figure 2.

Click here to Download Table S1

Journal of Cell Science • Supplementary information

Movies

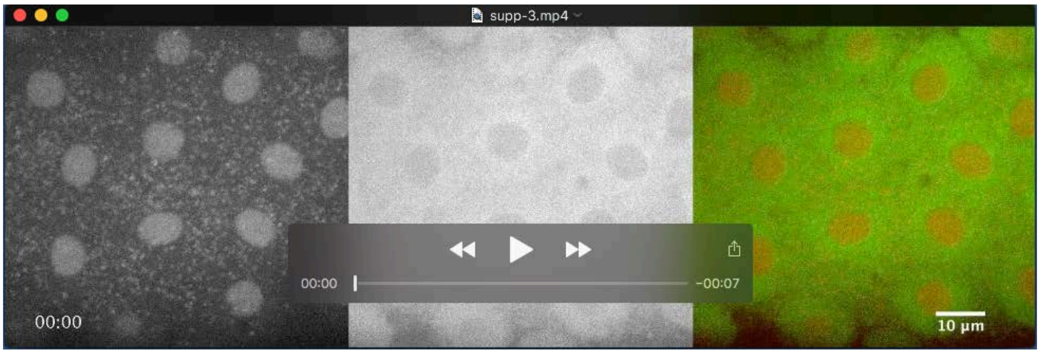

Movie 1. Localization of Mora-GFP in an embryo expressing Histone 2AV-RFP. The embryo is progressing through cycle 11; the left and middle channels show Histone-RFP and Mora-GFP respectively; in the merged channels (right panel) Histone-RFP is red and Mora green. Note that Mora-GFP is enriched in the perinuclear area during interphase and in the mitotic spindle throughout mitosis. Stills of this movie are shown in Figure 1A. Scale bar, 10 µm.

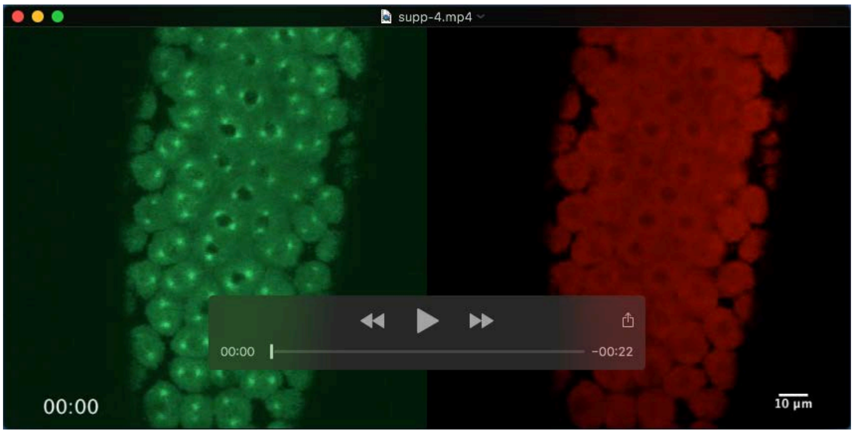

Movie 2. Localization of injected Alexa633-labelled anti-Mora antibodies in an embryo expressing α-Tubulin-GFP. The embryo is progressing from interphase of cycle 10 to interphase of cycle12. α-Tubulin-GFP is green and Alexa633-labelled Mora red. Note that the fluorescence of spindle-associated anti-Mora decreases concomitant with antibody-induced spindle shortening. Stills of this movie are shown in Figure 1B.

Journal of Cell Science • Supplementary information

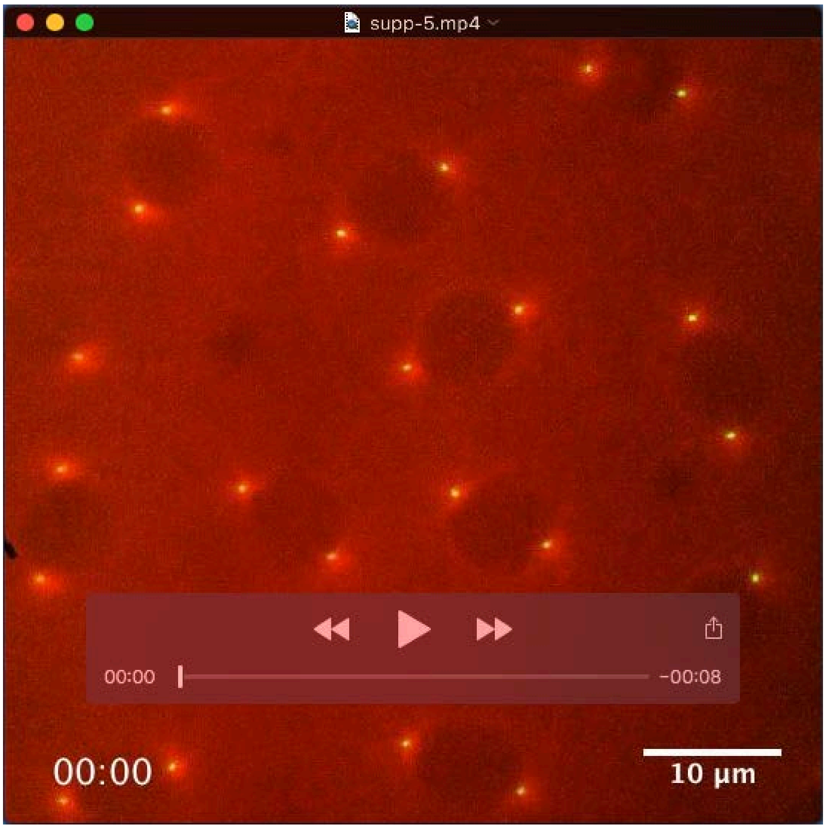

Movie 3. Mitotic divisions in a YFP-Asl expressing embryo simultaneously injected with rhodamine-labeled Tubulin and BSA (mock control). Imaging begins before NEB and shows the embryo progressing through cycle 11 with very well organized spindles; centrosomes are green and MTs red. Stills of this movie are shown in Figure 4A. Scale bar, 10 µm.

Journal of Cell Science • Supplementary information

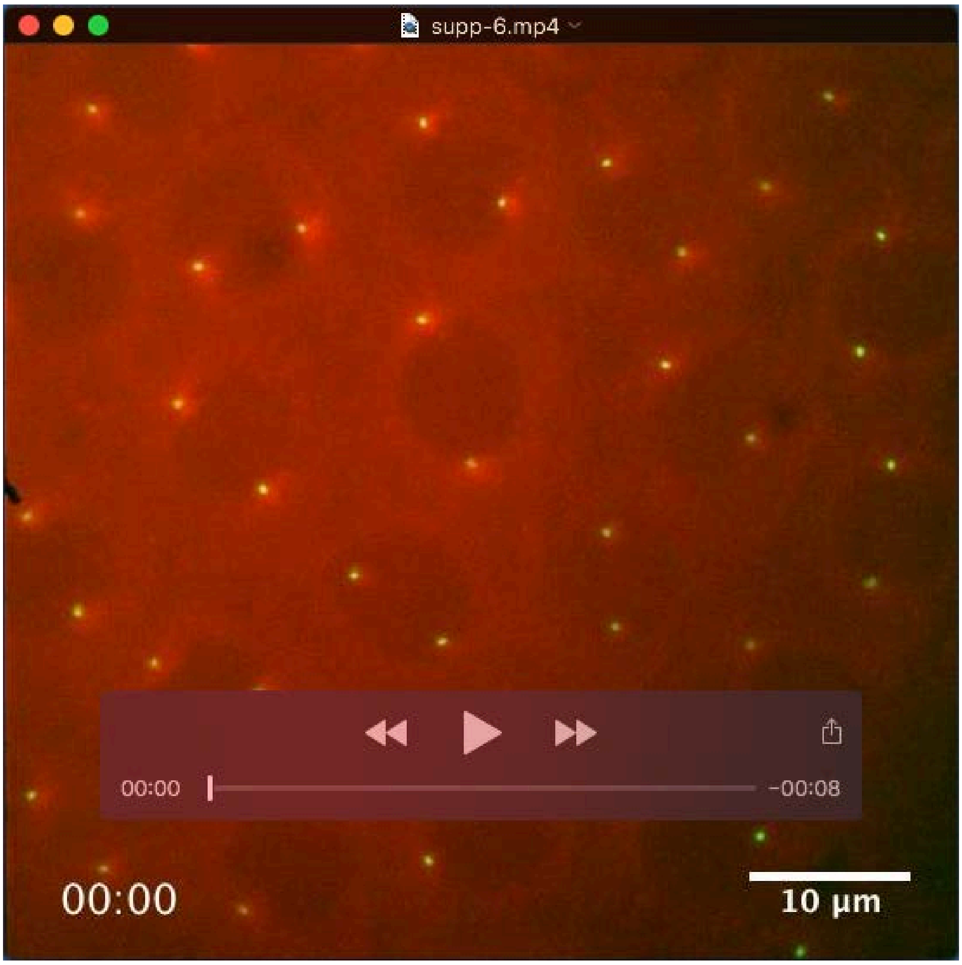

Movie 4. Mitotic divisions in a YFP-Asl expressing embryo simultaneously injected with rhodamine-labeled Tubulin and anti-Mora antibodies. Imaging begins before NEB and show the embryo progressing through cycle 11; centrosomes are green and MTs red. Note that the spindles close to the site of injection fail to form properly and exhibit severely disorganized clusters of short MTs. Stills of this movie are shown in Figure 4B. Scale bar, 10 µm.

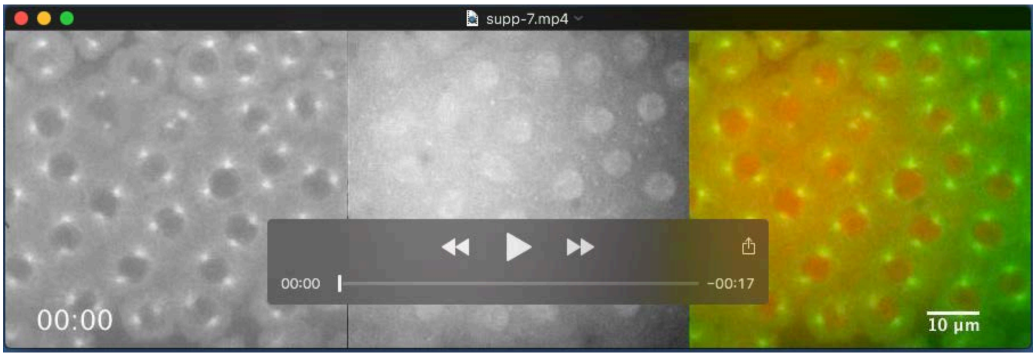

Movie 5. Mitotic divisions in an embryo co-expressing α-Tubulin-GFP and Histone-RFP, simultaneously injected with anti-Mora antibodies and rhodamine tubulin. α-Tubulin-GFP is green and His-RFP red; rhodamine Tubulin is also red and serves to mark antibody diffusion. The embryo is progressing through cycle 12 from prophase to telophase. Note that the antibody-induced short and disorganized spindles are not able to mediate chromosome segregation, resulting in abnormal mitotic products that sink into the embryo interior. Stills of this movie are shown in Figure C. Scale bar, 10 µm.

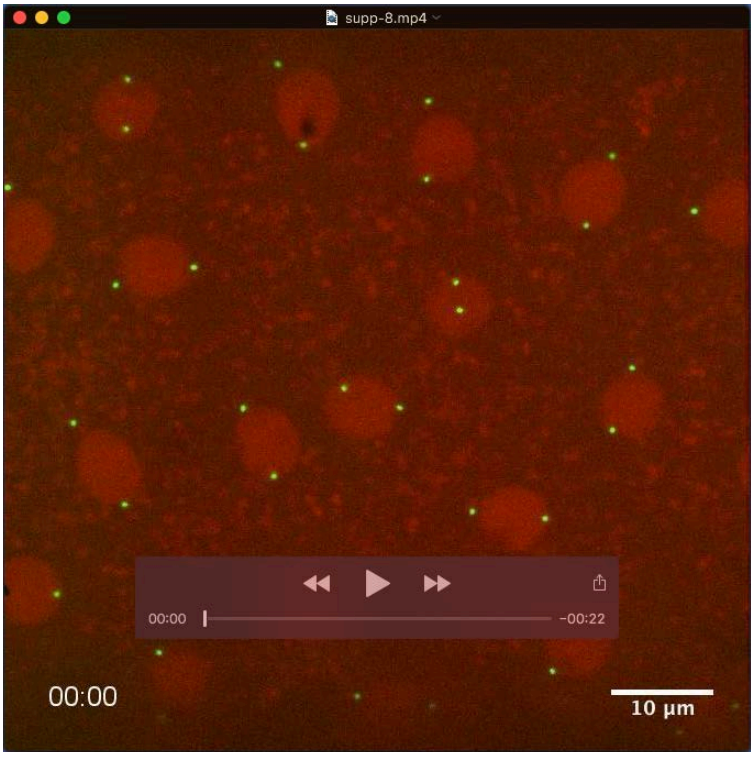

Movie 6. Mitotic divisions in a BSA-injected (mock control) embryo expressing YFP-Asl and Histone-RFP. Imaging begins before NEB and progresses from the interphase of cycle 11 to the interphase of cycle 13; centrosomes green, and chromatin red. Stills of this movie are shown in Figure S2. Scale bar, 10 µm.

Journal of Cell Science • Supplementary information

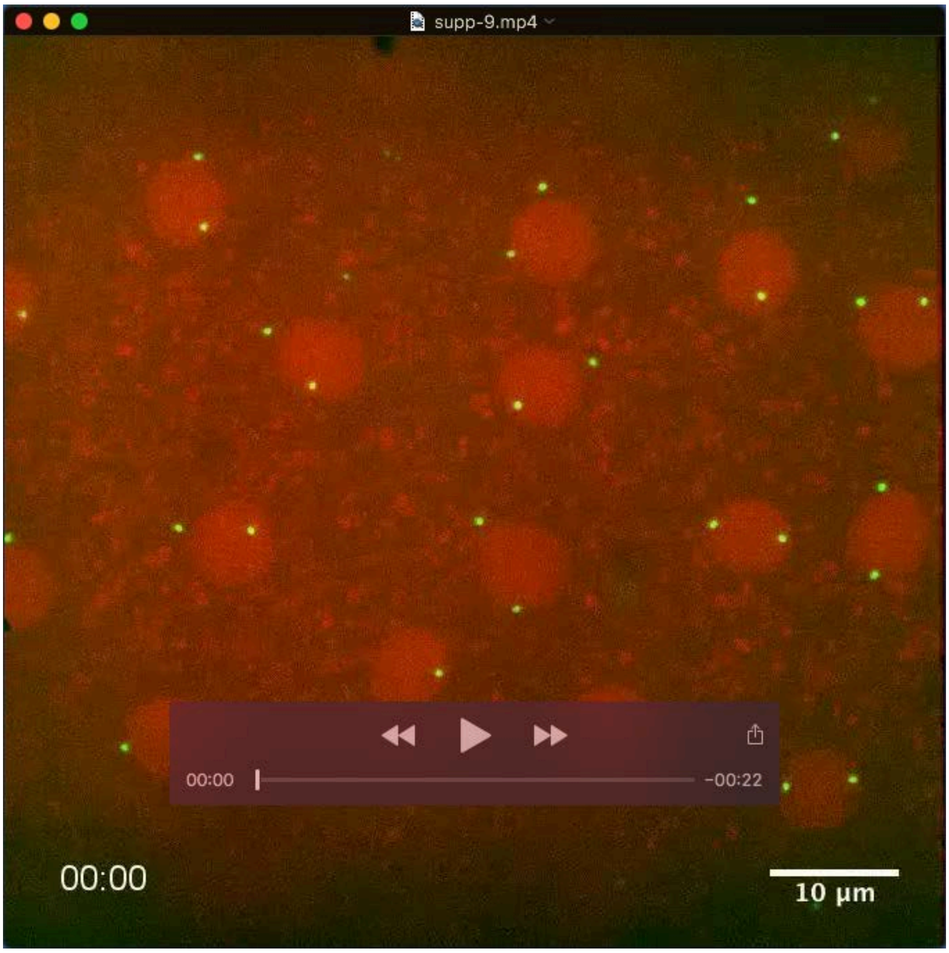

Movie 7. Mitotic divisions in an anti-Mora injected embryo expressing YFP-Asl and Histone-RFP. Imaging begins before NEB and progresses from the interphase of cycle 11 to the interphase of cycle 13; centrosomes are green, and chromatin red. Note that anti-Mora injection does not result in clear cases of centrosome overduplication. Stills of this movie are shown in Figure S2. Scale bar, 10 µm.

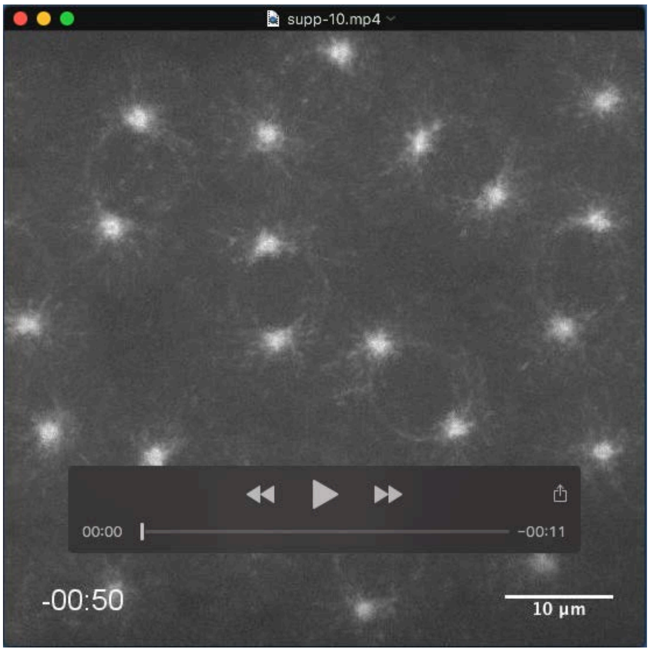

Movie 8. Mitotic divisions in a BSA-injected (mock control) embryo expressing Eb1-GFP. Imaging begins before NEB and shows a mitotic division of cycle 11 from prophase to telophase. Note that Eb1-GFP exhibits comet-like projections, with Eb1 concentrated in most distal tip of the growing MT. Stills of this movie are shown in Figure 4F. Scale bar, 10 µm.

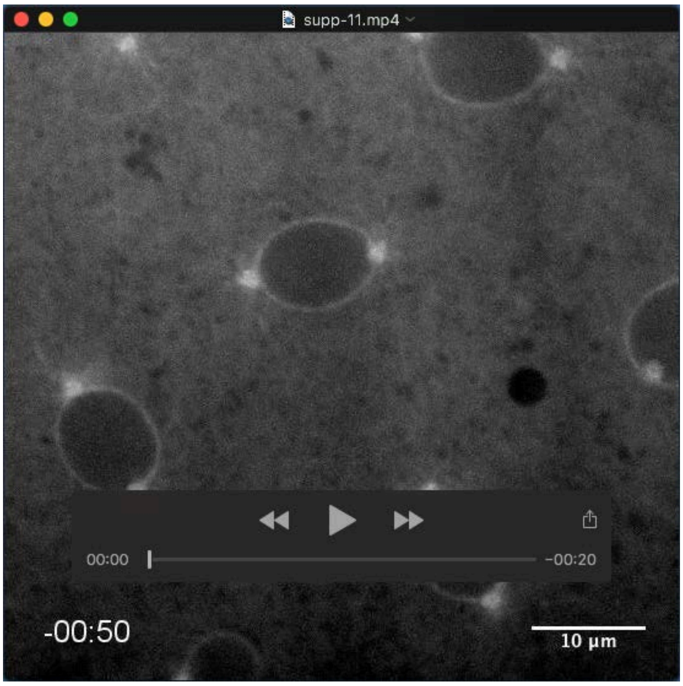

Movie 9. Mitotic divisions in an anti-Mora injected expressing Eb1-GFP. Imaging begins before NEB, shows a mitotic division of cycle 11 from prophase to telophase. Note that while Eb1-GFP is loaded at center of asters in interphase like in control (movie 8). However, following NEB, Eb1-GFP recruitment at growing MT plus ends is dramatically delayed and distinct comet tracks are lacking. Stills of this movie are shown in Figure 4G.

Journal of Cell Science • Supplementary information
